# INTRATHECAL BACLOFEN FOR NEUROFIBROMATOSIS RELATED SPINAL CORD INJURY WITH SPASTICITY – A CASE REPORT

**DOI:** 10.2340/jrm-cc.v7.25912

**Published:** 2024-08-13

**Authors:** Carl O’BRIEN, Jacqui STOWE, Michael O’CONNOR, Jacinta MORGAN, Paul MURPHY, Darren RODDY, Kirk LEVINS

**Affiliations:** 1 National Rehabilitation Hospital, Rochestown Avenue, Dun Laoghaire Co; 2 Royal College of Surgeons; 3 St Vincent’s University Hospital; 4 Department of Medicine, University College Dublin, Dublin, Ireland

**Keywords:** neurofibromatosis 1, spinal cord injury, spasticity, intrathecal baclofen

## Abstract

This case presents a 35-year-old male with spinal predominant neurofibromatosis-1 who developed an incomplete spinal cord injury (C3 Asia C) which did not improve despite urgent decompressive surgery for multiple cervical neurofibromas. This report outlines a novel indication for intrathecal baclofen. The patient suffered from lower limb weakness with severe spasticity and required assistance of at least two for all activities. The aim of rehabilitation was to improve overall tone while focusing on independence, mobility and quality of life. After a successful trial of intrathecal baclofen, he underwent implantation of an intrathecal baclofen pump. The dose was gradually increased while he received a progressive programme of stretching and functional rehabilitation therapy. After 6 weeks, his MAS had improved to 1–2/4 and he had progressed to independent transfers, independence for most activities of daily living and was able to discharge to his family home with minimal support.

## LAY ABSTRACT

Neurofibromatosis type 1 is a rare disorder causing various skin, skeletal, and neurological issues, including spinal cord injury due to tumours and severe scoliosis. Current treatment is supportive, with surgery sometimes needed for acute injuries. Spasticity, a common complication, leads to functional impairments and reliance on medications with side effects. Intrathecal baclofen pumps offer a targeted solution, delivering baclofen directly into the spine to reduce side effects. We present a case where a gentleman with neurofibromatosis-1-related spinal cord injury received intrathecal baclofen, coupled with intensive rehabilitation, to enhance comfort and daily independence. This case showcases the potential of intrathecal baclofen in managing spasticity in neurofibromatosis-1-related spinal cord disease.

Neurofibromatosis type 1 (NF1) is a rare autosomal dominant disorder characterized by various skin, skeletal, and neurological lesions, including the development of benign and malignant tumours, notably benign neurofibromas (1). A serious complication of NF1 is direct injury to the spinal cord, resulting from compression by tumours intrinsic and extrinsic to the thecal membrane, and/or severe scoliosis. Treatment is mainly supportive, but surgery may be necessary for acute spinal cord injury with neurological impairment and spasticity due to compressive features on imaging (2).

Spasticity in neurofibromatosis spinal cord disease is similar to other spinal cord lesions, leading to an unopposed stretch response within the musculo-spinal reflex pathway below the lesion level. Severe spasticity can cause significant functional impairments, and patients may develop permanent tendon shortening, infections, arthropathy, and pressure sores. Many are bedridden, relying on high doses of oral baclofen or other anti-spasmodic drugs for symptom relief. However, systemic use of baclofen can result in adverse effects such as fatigue, impaired cognition, nausea, and constipation due to its action on GABA receptors in the brain and autonomic nervous system (3).

Intrathecal baclofen (ITB) pumps provide a more targeted delivery of baclofen, reducing side effects and increasing efficacy. ITB requires surgical implantation of a programmable pump and catheter to deliver baclofen directly into the intrathecal space, and it is typically reserved for patients with severe spinal cord disease-related spasticity who have not responded adequately to oral agents and therapy.

In this case, a gentleman with incomplete tetra-paresis and severe spasticity related to spinal cord injury from neurofibromas was admitted for inpatient rehabilitation. He received ITB along with intensive interdisciplinary rehabilitation to improve his comfort, mobility, and independence in daily activities. This represents a unique indication for ITB in the management of NF1-related spinal cord disease spasticity.

## PATIENT INFORMATION

**Fig. 1:**
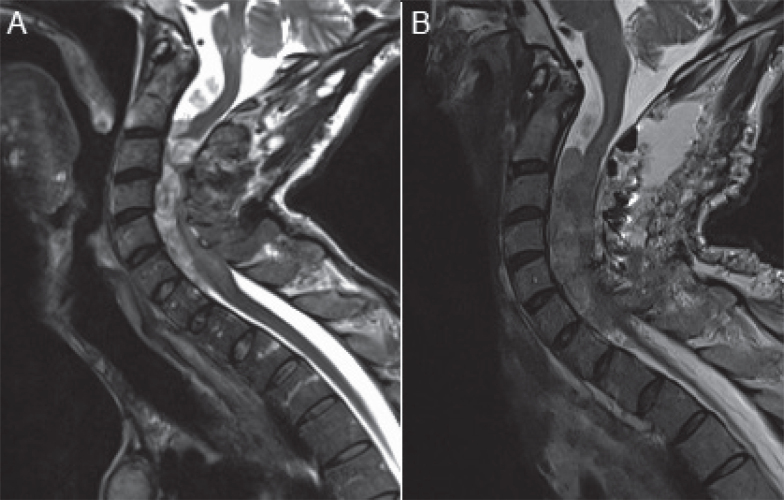
T2 weighted sagittal views of his cervico-thoracic spine. Image (A) is pre-operative and exhibits multiple intramedullary and extramedullary neurofibromas exerting pressure on the cervical cord. Image (B) is post-perform redo cervical laminoplasty and resection of intradural C2, C3, C5 and C6 extramedullary lesions, with post-operative oedema still visible in the spinal canal.

A 35-year-old male patient presented to neurosurgical services with severe spastic tetraplegia that had gradually worsened over 3 months. He had been diagnosed with NF1 6 months prior, initially reporting subtle lower leg weakness and recurrent falls over the previous 2 years, which led him to have to leave his employment as a sales assistant. An MRI spine confirmed numerous intra and extradural neurofibromas causing compression of the spinal cord at multiple points from C1 to C6. He was admitted and received IV steroid therapy followed by an urgent laminotomy and laminoplasty from C1 to C5 and removal of 2 lesions at C1 and C2. Post-operatively he had ongoing symptoms and it was decided 2 weeks later to perform a further cervical laminoplasty and resection of intradural lesions at C2, C3 and C5 and an extramedullary lesion at C6 (Fig. 1). He received oral baclofen therapy and occupational and physiotherapy care focusing on splinting and passive stretching as well as a referral for specialist neurorehabilitation at the National Rehabilitation Hospital in Ireland.

He was admitted for inpatient specialist spinal cord injury rehabilitation 3 months following his most recent surgery. Despite oral baclofen therapy and regular passive stretching, he suffered from severe spasticity. His hips and knees were fixed in flexion and deviating to his left in typical “windswept” appearance. He could not lie flat; washing, dressing and attempting to sit out on a chair were extremely painful. He also had severe spasms in both legs that kept him awake at night and impaired his concentration during the day.

## CLINICAL FINDINGS

On neurological assessment, he was found to have an incomplete spinal cord injury at the level of C2 (ASIA impairment scale C). He had 4/5 power in most upper limb muscles, but 1/5 power at the finger abductors bilaterally. He had 1–2/5 power in his right lower limb myotomes and 1–3/5 power in the left lower limb myotomes, with better power distally and normal voluntary anal contraction, sacral sensation, and deep anal pressure sensation. The Modified Ashworth Score (MAS) demonstrated 3 out of 4 tone in both hips and 4 out of 4 tone in both knees and ankles, indicating severe spasticity. He had normal (0 out 4 tone) in shoulder and elbow range of movement with mildly increased tone at the wrist and fingers (1 out of 4 tone).

## DIAGNOSTIC ASSESSMENT

The severity of the patient’s spinal cord injury, spasticity and disability were assessed via interdisciplinary clinical assessment using the MAS, ASIA assessment, rehabilitation complexity scale and Barthel index. Hip X-ray was employed to rule out other pathologies such as heterotopic ossificans, routine laboratory tests, for example, FBC, U+E, TFTs, B12, Folate were used to help rule out concomitant disabling conditions.

## THERAPEUTIC INTERVENTION

**Fig. 2:**
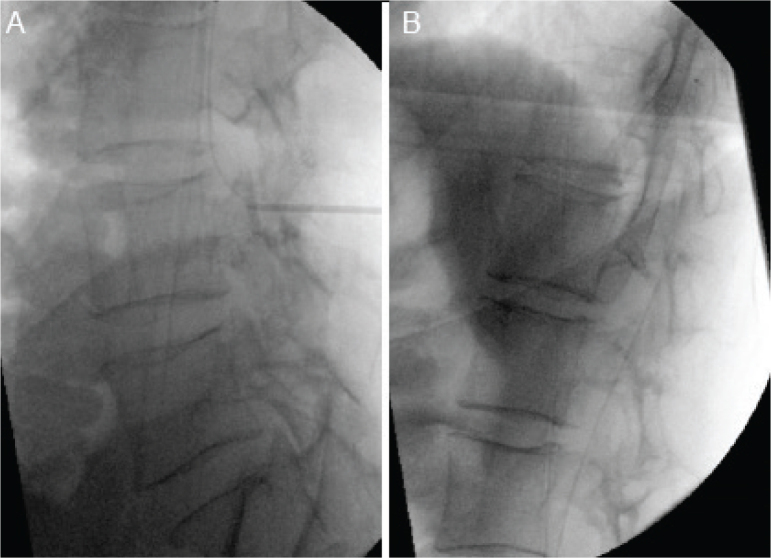
(A) Fluoroscopically guided lumbar puncture to deliver a trial of intrathecal baclofen. (B) Fluoroscopically guided insertion of intrathecal baclofen pump tunnelled catheter to the level of T6.

His care was managed by an interdisciplinary team, receiving a comprehensive treatment plan including trial of ITB +/- insertion of an ITB pump, graded increase in passive stretching and structured mobility rehabilitation (Fig. 2). The primary goals were to progress from requiring hoist transfer to independent transfers, to improve seating position and supports as well as achieve independence in most activities of daily living using adaptations to his environment and mobility aids.

On week 1 of admission, he received a trial of ITB: 50 micrograms of baclofen was injected intrathecally via lumbar puncture. After 1 h, he was re-assessed and found to have a significant improvement in his tone. Additionally, he reported that his painful spasms had resolved. He was subsequently admitted for ITB pump insertion the following week. A catheter was inserted into the lumbar cistern and guided fluoroscopically to the level of T6; this was connected via another tunnelled catheter to the pump, which was anchored in the abdominal wall. The reservoir in the pump was then filled with 1000 mcg/ml of baclofen; this relatively low concentration was employed to maximize cranial diffusion due to the high flow rate required to deliver the programmed dose. An incision was made in the right lower quadrant of the abdomen and a pouch was created in the anterior abdominal wall. The attached catheter was tunnelled along the sub-cutaneous layer adjacent to the lumbar spine and attached to the intrathecal catheter. Following wound closure, the device was programmed using the ITB pump Bluetooth™ programmer infusing of 100 mcg/day of baclofen. He was reviewed 6 h post-operatively and was found to have significantly improved tone with much reduced muscle spasms. He had several episodes of hypotension; however, he had no symptoms associated with this, and his blood pressure self-resolved.

Over the course of 5 weeks the daily baclofen infusion dose was gradually increased until a treatment plateau was reached as determined by the interdisciplinary team. While the dose was being increased, he received a graduated therapy programme. He received physiotherapy working on limb stretching as well as upper and lower limb strengthening. Functional therapeutic activities included transferring from bed to chair, using a power chair, dressing, toileting, and washing as well as functional kitchen and computer tasks. Optimal tone and function were achieved at a dose of 250 mcg/day but he continued to make gains during the course of his inpatient rehabilitation stay.

## FOLLOW-UP AND OUTCOMES

**Fig. 3:**
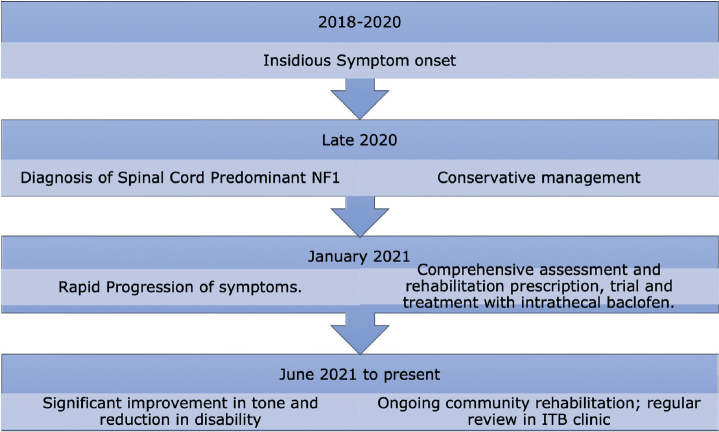
Timeline.

At week 7, post-insertion of the baclofen pump, he had progressed from a position of fixed flexion to a normal lying and sitting position. He progressed from requiring hoist transfers to self-transferring and safely navigating with a powered wheelchair. He could also return to one of his favourite hobbies: computer gaming. He reported a complete cessation of spasms and relief of pain, and he was able to stop regular opioid and paracetamol therapy. Similarly, he required no oral antispasmodic therapy. On power assessment: power in finger flexion increased from 1 to 4/5 bilaterally and power in both lower limbs showed slight improvement: 3/5 proximally and 4/5 distally. Tone in both upper limbs was completely normal (0/4 bilaterally) whereas tone in the lower limbs showed significant improvement (1 out of 4). Importantly, he progressed from requiring 24 hour nursing care and assistance for most activities to requiring only 1–2 hour of support a day which he could receive in his own home. For the timeline, see Fig. 3.

## DISCUSSION

This case highlights the potentially devastating effect of neurofibromatosis-induced spinal cord injury and a relatively novel modality of treatment: ITB via continuous infusion.

This patient suffers from NF, caused by a mutation in the NF1 gene on chromosome 17q11.2. 50% are familial with an autosomal dominant pattern; overall incidence is estimated between 1/36,000 and 1/40,000. It is characterized by cutaneous neurofibromas, café-au-lait spots, axillary freckling and skeletal dysplasia’s, as well as the development of both benign and malignant nervous system tumours. Spinal cord predominant NF1 is a relatively rare subset occurring in 5% of NF1 which is characterized by the preponderance of spinal neurofibromas both within and outside the thecal membrane (1). While cutaneous manifestations are cardinal feature of NF1, they occur less frequently in patients with spinal predominant disease (4). Common complications of spinal neurofibromatosis include scoliosis and kyphosis and spinal cord injury, which affected this gentleman from both internal and external compression (1).

As spinal neurofibromatosis is a rare condition, predicting who may be at risk of spinal cord compression and intervening in a timely manner remains challenging. The mainstay of treatment for neurofibroma-related spinal cord compression has been urgent steroid therapy and surgical decompression (1). The decision to proceed to surgical excision is predicated on patients symptoms; that is, paresis, spasticity, or autonomic dysfunction. Post-operative outcomes are not well understood, again owing to the rarity of the presentation. Ejerskov et al. performed one of the largest retrospective reviews of patients with NF1 treated for cervical cord compression – 11 patients across two large tertiary care centres were treated between 1996 and 2006. The most common presenting complaint was progressive quadriparesis. Clinical findings were reported heterogeneously, but five of the 11 patients were reported to have resolution of limb weakness post-operatively and nine of the 11 had improved or stable findings on long-term follow-up (5).

Like other causes of spinal cord injury, when a patient with spinal NF1 suffers from intractable tetra-paresis and spasticity, timely multidisciplinary rehabilitation is a vital component of care. This is best led by a rehabilitation physician in a spinal cord rehabilitation centre (6). Specialist spinal cord rehabilitation nursing will monitor and train patients and carers in adequate bowel, bladder management, positioning and skin protection as well as preventing and identifying complications such as autonomic dysreflexia. Physiotherapy is vital to engage and strengthen patient’s residual innervated muscle groups, training patients in safe transfers and mobility as well as enacting a programme of stretching to minimize the risks of permanent contracture. Occupational therapy addresses barriers in daily activities and may use splints for contracture risk. Specialists like respiratory therapists, social workers, psychology, and speech and language therapy are also involved, especially in patients with high spinal or swallowing disorders. Rehabilitation can be challenging for NF1 patients with psychiatric illness, learning delay, seizures, or painful skin lesions (6).

The primary medical treatment for spasticity is multimodal with oral agents often used in combination with passive stretching and other rehabilitation techniques (7). Initially, patients are treated with oral anti-spasmodic agents such as baclofen, tizanidine, dantrolene or benzodiazepines (8–10). Baclofen is probably the most commonly prescribed oral anti-spasmodic and as an analogue of γ-Aminobutyric acid (GABA) it inhibits the stimulatory function of GABA-B receptors at the spinal synapse of the muscle stretch reflex arc. This is the major pathway implicated in spasticity due to the absence of descending inhibitory signals from the cerebral cortex (8). Unfortunately, baclofen often has profound systemic effects when ingested orally which can include fatigue, bowel and bladder incontinence, cognitive impairment and susceptibility to seizures if withdrawn suddenly. Similar adverse effects are seen in all enteral anti-spasmodic agents (9).

ITB is an important treatment modality in many neurological conditions with associated hypertonia such as cerebral palsy, stroke, traumatic brain injury and, increasingly, spinal cord injury (10). It involves the insertion of a reservoir of baclofen and a programmable pump, usually subcutaneously in the abdominal wall with a subcutaneous catheter tunnelled to the lumbar spine and an attached intrathecal catheter up to the level of the distal to mid-thoracic spine. This allows a continuous and customizable infusion of baclofen into the thecal space – directly bathing the spinal cord. As a result, far lower doses of baclofen can have a much more significant effect on the musculo-spinal reflex pathway with a far lower risk of systemic side effects. This is a relatively invasive procedure compared to oral therapy and carries a risk of early adverse events such as post-operative bleeding, infection, or wound dehiscence as well as more chronic problems such as catheter problems, spinal granuloma formation and pump failure. It also involves regular follow-up to check for complications and refill the pump through percutaneous injection (10).

Despite the potential drawbacks, this intervention can often dramatically improve patients symptoms of spasticity and pain, improve their independence in the home and their ability to access and interact with the wider community (10). Interestingly, in this case, as well as significantly improving spasticity and pain levels, his upper and lower limb strength also appeared to have improved substantially on independent power assessment. This may be a result of gradual axonal regeneration post-excision of compressive neurofibromas, but may also have been helped by his improved ability to engage muscle groups during physiotherapy sessions while avoiding painful spasms. By improving spasticity symptoms and potentially effecting deficits, he was able to achieve significant measurable functional gains.

## PATIENT PERSPECTIVE

The baclofen pump allowed me to get moving and work with physiotherapy. This got me out of pain and boosted my independence, in particular my ability to transfer and use a wheelchair, which was the most important thing for me.
